# Supplementary material for: Transcriptomic and Metabolomic Profile Analysis of Muscles Reveals Pathways and Biomarkers Involved in Flavor Differences between Caged and Cage-Free Chickens
Source: Foods. 2022 Sep 17;11(18):2890. doi: 10.3390/foods11182890 (PMC9498551; doi:10.3390/foods11182890)
Supplement: Supplementary file 1 [file foods-11-02890-s001.zip › Supplementary file S1ú║Primer information.docx]

TableS1. Primer information for qPCR verification

| Primer | Sequence |
| --- | --- |
| GAPDH-F | CCCCCATGTTTGTGATGGGT |
| GAPDH-R | TGATGGCATGGACAGTGGTC |
| NR4A3-F | AGACGCCGTAACAGATGTCA |
| NR4A3-R | TCGTGAATAGTCGAGCTCCC |
| FABP3-F | CCGGCCTGACCAAACCC |
| FABP3-R | TGCCTCCATCTAGCTTGACCA |
| GLS2-F | TTCCTAATGTCATGGGCTTG |
| GLS2-R | CTTGACCCGCTGATCACCA |
| COL3A1-F | CTTACCCGGTAGCAACGGACT |
| COL3A1-R | CTGGGCCTCCATCTTGACCAC |
| FBN1-F | TCTTTCCGCTGTGAATGTCC |
| FBN1-R | CACTACATTCCTGCAAGTTCC |
