# Supplementary material for: Transcriptomic and Metabolomic Profile Analysis of Muscles Reveals Pathways and Biomarkers Involved in Flavor Differences between Caged and Cage-Free Chickens
Source: Foods. 2022 Sep 17;11(18):2890. doi: 10.3390/foods11182890 (PMC9498551; doi:10.3390/foods11182890)
Supplement: Supplementary file 1 [file foods-11-02890-s001.zip › Supplementary file S2. Data analysis of sensory evaluation..pdf]

Table 1. Linear regression analysis between age, sex, rearing systems and breast muscle aroma intensity (n=82)

	Unstandardized Coefficients		Standardized Coefficients	t	p	VIF	R^2	Adjusted R^2	F
	B	Standard error	$Beta$						
Constant	4.532	0.901	-	5.027	0.000**	-	0.016	-0.022	F(3,78)=0.431,p=0.731
Age	-0.000	0.031	-0.001	-0.005	0.996	1.056			
SEX	-0.152	0.238	-0.074	-0.640	0.524	1.056			
SYS	-0.195	0.210	-0.104	-0.927	0.357	1.000			

Dependent variable: Aroma intensity; D-W value:2.034; *P<0.05; ** P<0.01.

Table 2. Linear regression analysis between age, sex, rearing systems and breast muscle initial impression of juiciness (n=82)

	Unstandardized Coefficients		Standardized Coefficients	t	p	VIF	R^2	Adjusted R^2	F
	B	Standard error	$Beta$						
Constant	3.910	0.741	-	5.276	0.000**	-	0.013	-0.025	F(3,78)=0.331,p=0.803
Age	-0.024	0.026	-0.107	-0.922	0.359	1.056			
SEX	-0.006	0.195	-0.003	-0.030	0.976	1.056			
SYS	0.049	0.173	0.032	0.282	0.779	1.000			

Dependent variable: Initial impression of juiciness; D-W value:1.590; *P<0.05; ** P<0.01.

Table 3. Linear regression analysis between age, sex, rearing systems and breast muscle first bite (n=82)

	Unstandardized Coefficients		Standardized Coefficients	t	p	VIF	R^2	Adjusted R^2	F
	B	Standard error	$Beta$						
Constant	3.905	0.743	-	5.257	0.000**	-	0.031	-0.006	F(3,78)=0.845,p=0.473
Age	-0.007	0.026	-0.030	-0.264	0.792	1.056			
SEX	-0.038	0.196	-0.022	-0.196	0.845	1.056			
SYS	-0.268	0.173	-0.172	-1.548	0.126	1.000			

Dependent variable: First bite ; D-W value:2.215; *P<0.05; ** P<0.01.

Table 4. Linear regression analysis between age, sex, rearing systems and breast muscle sustained impression of juiciness (n=82)

	Unstandardized Coefficients		Standardized Coefficients	t	p	VIF	R^2	Adjusted R^2	F
	B	Standard error	*Beta*						
Constant	2.874	0.704	-	4.083	0.000**	-	0.047	0.010	$F(3,78)=1.278, p=0.288$
Age	0.035	0.024	0.163	1.431	0.157	1.056			
SEX	-0.057	0.186	-0.035	-0.307	0.760	1.056			
SYS	-0.220	0.164	-0.148	-1.336	0.185	1.000			

Dependent variable: sustained impression of juiciness ; D-W value:2.282; *P<0.05; ** P<0.01.

Table 5. Linear regression analysis between age, sex, rearing systems and breast muscle fiber and overall tenderness (n=82)

	Unstandardized Coefficients		Standardized Coefficients	t	p	VIF	R^2	Adjusted R^2	F
	B	Standard error	*Beta*						
Constant	5.785	0.673	-	8.591	0.000**	-	0.093	0.058	$F(3,78)=2.654, p=0.054$
Age	-0.035	0.023	-0.165	-1.493	0.140	1.056			
SEX	0.284	0.177	0.177	1.599	0.114	1.056			
SYS	-0.317	0.157	-0.218	-2.018	0.047*	1.000			

Dependent variable: Muscle fiber and overall tenderness; D-W value:1.854 ; *P<0.05; ** P<0.01.

Table 6. Linear regression analysis between age, sex, rearing systems and breast muscle amount of connective tissue(n=82)

	Unstandardized Coefficients		Standardized Coefficients	t	p	VIF	R^2	Adjusted R^2	F
	B	Standard error	*Beta*						
Constant	1.190	0.381	-	3.123	0.003**	-	0.018	-0.020	$F(3,78)=0.481, p=0.696$
Age	0.004	0.013	0.031	0.266	0.791	1.056			
SEX	0.034	0.100	0.039	0.339	0.735	1.056			
SYS	-0.098	0.089	-0.123	-1.097	0.276	1.000			

Dependent variable: Amount of connective tissue; D-W value:2.062 ; *P<0.05; ** P<0.01.

Table 7. Linear regression analysis between age, sex, rearing systems and breast muscle overall flavor intensity (n=82)

	Unstandardized Coefficients		Standardized Coefficients	*t*	*p*	VIF	R^2	Adjusted R^2	*F*
	B	Standard error	*Beta*						
Constant	4.234	0.792	-	5.344	0.000**	-	0.019	-0.019	*F* (3,78)=0.503,*p*=0.681
Age	-0.013	0.027	-0.055	-0.476	0.635	1.056			
SEX	0.238	0.209	0.132	1.142	0.257	1.056			
SYS	0.073	0.185	0.044	0.396	0.693	1.000			

Dependent variable: overall flavor intensity ; D-W value:2.322; *P<0.05; ** P<0.01.

Table 8. Linear regression analysis between age, sex, rearing systems and breast muscle overall off-flavor intensity (n=82)

	Unstandardized Coefficients		Standardized Coefficients	*t*	*p*	VIF	R^2	Adjusted R^2	*F*
	B	Standard error	*Beta*						
Constant	3.261	0.674	-	4.839	0.000**	-	0.046	0.010	*F* (3,78)=1.263,*p*=0.293
Age	-0.021	0.023	-0.102	-0.902	0.370	1.056			
SEX	-0.138	0.178	-0.088	-0.779	0.439	1.056			
SYS	0.220	0.157	0.154	1.396	0.167	1.000			

Dependent variable: overall off-flavor intensity ; D-W value:2.310; *P<0.05; ** P<0.01.

Table 9. Linear regression analysis between age, sex, rearing systems and leg muscle aroma intensity (n=82)

	Unstandardized Coefficients		Standardized Coefficients	t	p	VIF	R^2	Adjusted R^2	F
	B	Standard error	*Beta*						
Constant	5.729	0.786	-	7.290	0.000**	-	0.056	0.020	$F(3,78)=1.544, p=0.210$
SEX	-0.167	0.207	-0.091	-0.807	0.422	1.056			
Age	0.004	0.027	0.018	0.162	0.872	1.056			
SYS	-0.366	0.183	-0.219	-1.995	0.050*	1.000			

Dependent variable: Aroma intensity; D-W value:1. 426; *P<0.05; ** P<0.01.

Table 10. Linear regression analysis between age, sex, rearing systems and leg muscle initial impression of juiciness (n=82)

	Unstandardized Coefficients		Standardized Coefficients	t	p	VIF	R^2	Adjusted R^2	F
	B	Standard error	*Beta*						
Constant	5.085	0.812	-	6.265	0.000**	-	0.044	0.007	$F(3,78)=1.191, p=0.319$
SEX	-0.247	0.214	-0.132	-1.156	0.251	1.056			
Age	-0.006	0.028	-0.023	-0.201	0.841	1.056			
SYS	-0.268	0.189	-0.157	-1.416	0.161	1.000			

Dependent variable: Initial impression of juiciness; D-W value:1.620; *P<0.05; ** P<0.01.

Table 11. Linear regression analysis between age, sex, rearing systems and leg muscle first bite (n=82)

	Unstandardized Coefficients		Standardized Coefficients	t	p	VIF	R^2	Adjusted R^2	F
	B	Standard error	*Beta*						
Constant	3.856	0.908	-	4.246	0.000**	-	0.009	-0.029	$F(3,78)=0.240, p=0.868$
SEX	0.016	0.239	0.008	0.068	0.946	1.056			
Age	0.014	0.031	0.053	0.458	0.648	1.056			
SYS	0.146	0.212	0.078	0.690	0.492	1.000			

Dependent variable: First bite ; D-W value:2.215; *P<0.05; ** P<0.01.

Table 12. Linear regression analysis between age, sex, rearing systems and leg muscle sustained impression of juiciness (n=82)

	Unstandardized Coefficients		Standardized Coefficients	t	p	VIF	R^2	Adjusted R^2	F
	B	Standard error	$Beta$						
Constant	5.719	0.676	-	8.460	0.000**	-	0.080	0.045	F (3,78)=2.272, p=0.087
SEX	0.102	0.178	0.064	0.573	0.568	1.056			
Age	-0.027	0.023	-0.129	-1.157	0.251	1.056			
SYS	-0.366	0.158	-0.252	-2.319	0.023*	1.000			

Dependent variable: sustained impression of juiciness ; D-W value:1.911 ; *P<0.05; ** P<0.01.

Table 13. Linear regression analysis between age, sex, rearing systems and leg muscle fiber and overall tenderness (n=82)

	Unstandardized Coefficients		Standardized Coefficients	t	p	VIF	R^2	Adjusted R^2	F
	B	Standard error	$Beta$						
Constant	4.570	0.933	-	4.901	0.000**	-	0.006	-0.032	F (3,78)=0.157, p=0.925
SEX	0.029	0.246	0.014	0.118	0.906	1.056			
Age	0.001	0.032	0.005	0.045	0.964	1.056			
SYS	-0.146	0.218	-0.076	-0.672	0.503	1.000			

Dependent variable: Muscle fiber and overall tenderness; D-W value:1.877 ; *P<0.05; ** P<0.01.

Table 14. Linear regression analysis between age, sex, rearing systems and leg muscle amount of connective tissue(n=82)

	Unstandardized Coefficients		Standardized Coefficients	t	p	VIF	R^2	Adjusted R^2	F
	B	Standard error	$Beta$						
Constant	1.019	0.583	-	1.747	0.085	-	0.017	-0.021	F (3,78)=0.445, p=0.721
SEX	0.027	0.154	0.020	0.177	0.860	1.056			
Age	0.021	0.020	0.122	1.057	0.294	1.056			
SYS	0.024	0.136	0.020	0.179	0.858	1.000			

Dependent variable: Amount of connective tissue; D-W value:1.955 ; *P<0.05; ** P<0.01.

Table 15. Linear regression analysis between age, sex, rearing systems and leg muscle overall flavor intensity (n=82)

	Unstandardized Coefficients		Standardized Coefficients	t	p	VIF	R^2	Adjusted R^2	F
	B	Standard error	$Beta$						
Constant	7.050	0.815	-	8.652	0.000**	-	0.123	0.089	F(3,78)=3.648, p=0.016
SEX	-0.459	0.215	-0.233	-2.138	0.036*	1.056			
Age	-0.026	0.028	-0.102	-0.937	0.352	1.056			
SYS	-0.390	0.190	-0.218	-2.052	0.044*	1.000			

Dependent variable: overall flavor intensity ; D-W value:2.301; *P<0.05; ** P<0.01.

Table 16. Linear regression analysis between age, sex, rearing systems and leg muscle overall off-flavor intensity (n=82)

	Unstandardized Coefficients		Standardized Coefficients	t	p	VIF	R^2	Adjusted R^2	F
	B	Standard error	$Beta$						
Constant	2.349	0.785	-	2.992	0.004**	-	0.019	-0.019	F(3,78)=0.505, p=0.680
SEX	0.164	0.207	0.091	0.791	0.431	1.056			
Age	-0.009	0.027	-0.038	-0.329	0.743	1.056			
SYS	0.171	0.183	0.104	0.932	0.354	1.000			

Dependent variable: overall off-flavor intensity ; D-W value:1.703; *P<0.05; ** P<0.01.
